# Bilayered Films Based on Novel Polymer Derivative for Improved Ocular Therapy of Gatifloxacin

**DOI:** 10.1155/2014/297603

**Published:** 2014-01-02

**Authors:** Naval Dinesh Aher, Hema Ajit Nair

**Affiliations:** Department of Pharmaceutics, Bombay College of Pharmacy, Kalina, Santacruz East, Mumbai 400 098, India

## Abstract

*Context*. Thiomers could prove to be suitable mucoadhesives for fabrication of ocular inserts. *Objective*. The study intends to explore the application of thiolated sodium alginate (TSA) to the preparation of bilayered ocular inserts of gatifloxacin. *Methods*. Cysteine moieties were grafted onto sodium alginate (SA) and the resultant thiomer was characterized for relevant physicochemical properties. Bilayered inserts were fabricated with a mucoadhesive immediate release layer composed of either SA or TSA and a sustained release layer composed of acrylates. Films were prepared by solvent evaporation and evaluated for mechanical properties, drug content, and *in vitro* release. *Results and Discussion*. The synthesized TSA possessed 248.80 ± 49.7 **μ**mol thiol groups/gm and its solutions thickened on standing due to disulphide bridging. Its films showed improved mucoadhesion and also a strikingly beneficial property of resisting erosion and remaining as a hydrated adhesive layer for the duration of drug release. The bilayered films were found to be flexible, with good folding endurance, uniform thickness, and appropriate drug content, and showed a release of about 80% of loaded gatifloxacin in 12 h. *Conclusion*. The study demonstrates promise in employing thiolated polymer in conjunction with acrylates for the design of ocular inserts for twice a day therapy with gatifloxacin.

## 1. Introduction

Drugs administered in traditional topical ophthalmic formulations such as aqueous eye drops have poor bioavailability due to rapid turnover of tears, reflex blinking and reflex tearing, lachrymal drainage to the nose, and limited permeability of the cornea. To achieve therapeutic levels frequent instillations of the drug is required, which leads to low patient compliance. In addition, the drug level in the tear film is pulsed, with an initial period of overdosing, followed by a longer period of underdosing [1, 2]. Consequently, numerous novel ophthalmic drug delivery systems have been developed to achieve improved bioavailability of drugs, for example, *in situ *gelling polymers, micro/nanoparticles, lipo/niosomes, and ocular inserts [3]. Ocular inserts, which are solid devices placed in the cul-de-sac of the eye, offer significant advantages in comparison to other liquid formulations. By design of devices with prolonged retention in the eye coupled with controlled release of the active agent, an effective drug concentration in the eye can be ensured over a time period. Dosing of the drugs is also more accurate and the risk of systemic side effects is decreased. Furthermore, solid devices have an increased shelf life and the presence of additives such as preservatives is not required [4].

Despite all of the above advantages ocular inserts have enjoyed only limited success in ocular therapy. One disadvantage is the foreign body sensation these solid devices are likely to cause in the patients eye. Inserts without appropriate mucoadhesive properties can move around on the ocular surface, causing further irritation, and might easily be lost. The erosion and/or disintegration of soluble inserts into smaller pieces results in occasional blurring of vision. Further, in case of soluble inserts, change in viscosity of tears due to dissolution of polymers may lead to interference with blinking [3, 5].

The prerequisite for successful delivery of a drug across the ocular mucosa is the use of adhesive dosage forms which can remain anchored to the ocular mucosa for designated time, that is, the use of muco/bioadhesive polymers.

Mucoadhesion of most polymers involves a combination of surface and diffusional phenomena that contribute to the formation of semipermanent interchain bridges between the polymer and the mucosal surface. The various proposed mechanisms of mucoadhesion include lowering of interfacial tension, interpenetration of the mucoadhesive polymer and mucin, formation of an electrical double layer at the adhesive/mucin interface, and formation of hydrogen bonds and/or van der Waals' forces of attraction [6].

Novel classes of polymers that are capable of intimate interaction with the mucosal surface through formation of stronger covalent bonds are under investigation. One such class of polymers are the thiolated polymers or thiomers. Thiomer derivatives are synthesized by forming conjugates of polymers with thiol bearing moieties such as L-cysteine and iminothiolane. Thiomers exhibit improved mucoadhesive properties due to formation of covalent bond between thiol group of polymer and cysteine rich subdomains of mucus glycoprotein. Furthermore, thiolation is reported to impart other beneficial properties such as enzyme inhibitory potential, permeation enhancing effect, and improved cohesiveness of matrices due to formation of intrapolymeric disulfide bonds at physiological pH which in turn can lead to improved release retardant properties. Thiolated polymers have been successfully investigated for various drug delivery systems such as matrix tablets, microparticulates, films, and liquid formulations for drug delivery via oral, buccal, ocular, nasal, and vaginal routes due to their beneficial properties [7, 8].

Alginate and alginic acid are the generic terms applied to a naturally occurring, commercially important family of hydrophilic linear unbranched polysaccharides. It contains varying proportions of *β*-d-mannuronic acid and *α*-l-guluronic acid extracted from a number of closely related species of brown seaweed. Sodium alginate (SA) is used in a variety of oral and topical pharmaceutical formulations. One of the reasons for its wide use in pharmaceutical formulations is its mucoadhesive and release retardant properties [9]. In particular, for ocular drug delivery, SA has been widely investigated as a carrier for small molecular weight compounds in the form of micro/nanoparticles and ocular inserts [10].

Polymethacrylates used in ophthalmic drug delivery include mainly Eudragit RL 100 (ERL) and Eudragit RS 100 (ERS) for fabrication of solid dosage forms like ocular films. The ERS film is less permeable to water than ERL; hence, films of varying permeability can be obtained by mixing the two types together [11, 12]. Micro/nanoparticulate formulations ERS and ERL with different drugs like acyclovir, acetazolamide, ibuprofen, and gentamicin are also reported for prolonged drug delivery to the eye [13].

Gatifloxacin, an antibiotic of the fourth-generation fluoroquinolone family, inhibits the bacterial enzymes DNA gyrase and topoisomerase IV. It is bactericidal with an improved antibacterial spectrum, particularly against resistant *staphylococcus *and *streptococcus *pathogens, compared to older fluoroquinolones. Gatifloxacin is available as tablets and as various aqueous solutions for intravenous and ophthalmic therapy. Gatifloxacin is administered intraocularly as a 0.3% (w/v) solution as 1 drop every 2 h on the first two days of therapy into the affected eye(s) while awake, up to eight times per day, followed by 1 drop upto four times per day while awake during the next 5 days [14]. Thus, to maintain desired levels, frequent instillation of drops is required. Hence, gatifloxacin was selected as model drug for sustained delivery to the eye.

The present study was aimed at developing a mucoadhesive ocular insert of gatifloxacin which would maintain effective drug concentration in the eye for prolonged periods of time. The system was designed as a bilayered insert with a mucoadhesive immediate release layer composed either of native or thiolated alginate and a sustained release layer prepared using acrylates.

## 2. Experimental 

### 2.1. Materials

Sodium alginate (Protanal LF 120 M) was obtained as a gift sample from Signet Chemical Corporation, Mumbai, India. Cysteine hydrochloride monohydrate, sodium borohydride, glycerine, and dibutyl phthalate (DBP) were purchased from S.D. fine chemicals, Mumbai, India. 1-ethyl-3-(3-dimethylaminopropyl) carbodiimide hydrochloride (EDAC), Ellman's regent (5,5-dithiobis (2-nitro benzoic acid), and artificial porcine mucin were purchased from Sigma-Aldrich. Gatifloxacin sesquihydrate was gifted by FDC Pvt. Ltd. Mumbai, India. Eudragit RL 100 and Eudragit RS 100 were procured from Evonik Degussa Pvt. Ltd. Dialysis membrane (cut off size M.W 12–16 KDa.) was purchased from Himedia, Mumbai, India. All other chemicals and reagents used in the study were of analytical grade.

### 2.2. Methods

#### 2.2.1. Synthesis of Alginate-Cysteine Conjugate

Sodium alginate-cysteine conjugate (TSA) was synthesized according to a method described previously by Bernkop-Schnürch et al. [15]. SA was hydrated in distilled water, followed by addition of 1-ethyl-3-(3-dimethylaminopropyl) carbodiimide hydrochloride (EDAC) at a final concentration of 100 mM to activate the carboxylic acid groups of the polymer. pH of the reaction medium was adjusted to 6.0 with 0.1 M HCl. The mixture was incubated for 45 min. At the end of incubation period, cysteine hydrochloride monohydrate in the weight ratio of 2 : 1 (polymer : cysteine) was added. pH was readjusted to 4.0 and reaction was continued for 3 h at room temperature under continuous stirring using a magnetic needle. The reaction was carried out in dark and the resulting alginate-cysteine conjugate was isolated by dialyzing at 10°C against 1 mM HCl for 24 h, followed by dialysis against 1 mM HCl containing 1% (w/v) NaCl for 48 h and then exhaustively against 1 mM HCl (pH 4.0). Control polymer was prepared in an identical manner except that EDAC was omitted. The polymer derivative in solution was directly used for estimation of thiol content and for casting of films.

### 2.3. Characterization of Sodium Alginate-Cysteine Conjugate

#### 2.3.1. Determination of the Thiol Group Content

The conjugate was characterized for free (unoxidized) as well as total (after reduction of all disulfide bonds) thiol content using Ellman's reagent (DTNB, 5,5-dithiobis (2-nitro benzoic acid). Here DTNB reacts with thiol group to release TNB^−^ ion which further ionizes to TNB^−2^. The divalent ion has a yellow colour that can be detected by visible light at 410 nm. The dialyzed polymer conjugate/control solution was diluted 10-fold with distilled water to result in a final polymer concentration of 2 mg/mL. To 250 *μ*L of the diluted conjugate solutions, 250 *μ*L of 0.5 M phosphate buffer pH 8.0 and 500 *μ*L of Ellman's reagent were added. The samples were incubated for 2 h at room temperature followed by measurement of absorbance of the resultant solutions at a wavelength of 410 nm by UV-visible spectrophotometry. The concentration of the thiol group was determined from a calibration curve obtained by treating L-cysteine in an identical manner.

#### 2.3.2. Determination of Total Thiol Content (after Reduction of All Disulfide Bonds)

Dialyzed polymer conjugate/control solution was diluted 10-fold with distilled water to result in a 2 mg/mL solution of polymer. To 250 *μ*L of this solution, 750 *μ*L of 0.05 M phosphate buffer pH 6.8 was added followed by 1 mL of a freshly prepared 4% (w/v) solution of sodium-borohydride for reduction of the disulfide bonds. The mixture was then incubated for 1 h at room temperature. Thereafter, 200 *μ*L of 3 M HCl was added and the reaction mixture was agitated for 10 min in order to destroy the remaining sodium borohydride. The solution was neutralized by addition of 1 mL 1 M phosphate buffer pH 8.5 followed by prompt addition of 100 *μ*L freshly prepared Ellman's reagent. Mixtures were incubated for 2 h at ambient conditions, absorbance was measured at 410 nm, and the total proportion of bound cysteine was calculated from a calibration curve prepared using L-cysteine.

#### 2.3.3. Fourier Transform Infrared Spectroscopy (FTIR)

For recording FTIR spectrum, the polymer was precipitated from the aqueous solution by addition of isopropyl alcohol and then air dried. About 2-3 mg of either SA or TSA was deposited on the surface of KBr pellets and FTIR spectra were recorded (Jasco FTIR 5300). The IR spectra were analyzed for evidence of amide bond formation.

#### 2.3.4. Rheological Studies

The TSA in solution after dialysis was diluted with distilled water to result in a 1% (w/v) solution of polymer. A 1% (w/v) solution of native SA was also prepared. The pH of the polymer solutions was adjusted to 7.0 with 1 M NaOH. Measurements were made using Brookfield LVT viscometer soon after preparation of the solutions and once after every 24 h for three days thereafter. Flow curves were recorded each time by applying continuously increasing shear rates at ambient temperatures. Rheological behaviour was elucidated using plots of RPM versus viscosity.

### 2.4. Preparation of Ocular Films

Initially, drug loaded films of mucoadhesive layer and sustained release layer were prepared and evaluated separately using solvent casting technique.

For preparation of the mucoadhesive layer of ocular inserts, sodium alginate (SA) or its thiolated derivative (TSA) were used. Both polymers were used as 2% (w/v) solution; while the native polymer was dissolved in distilled water, solution of polymer after dialysis was directly used in case of TSA. The plasticizer (glycerin, 30% (w/w) of polymer) was added and stirred for uniform mixing followed by addition of gatifloxacin. The resultant mixture was stirred, allowed to stand until all air bubbles disappeared, and then poured into a leveled polypropylene petridish and allowed to dry in an oven at 45°C for 12 h and a clear film was obtained.

Sustained release layer was cast using a mixture of Eudragit RL 100 and Eudragit RS 100 in 75 : 25 proportion, respectively, dissolved in acetone : ethanol (80 : 20). Dibutyl phthalate (DBP) (30% (w/w) of polymer) was added to polymer mixture as plasticizer followed by addition of gatifloxacin. This mixture was stirred on a magnetic stirrer and cast on mercury containing plates. The mucoadhesive films (SA or TSA) and sustained release films (Eudragit) were cut using a punch into circular inserts with dimensions of 78.5 mm^2^, containing 0.3 mg (loading dose) and 2.1 mg (maintenance dose) of gatifloxacin per film, respectively. The films were packed in aluminium foil and stored in a glass bottle at room temperature.

### 2.5. Evaluation of Ocular Films

#### 2.5.1. Thickness

Thickness of five uncut films was measured at five different places (centre and four corners) using Vernier Callipers and the average was determined.

#### 2.5.2. Folding Endurance

Folding endurance was determined by repeatedly folding and reopening the film at the same place till it broke or was folded up to 300 times, whichever happened earlier.

#### 2.5.3. Mechanical Properties

Tensile strength and elongation at break of films were measured using INSTRON tester. The film samples were attached to upper and lower grips of tester. The grip separation was set at 5 cm. The crosshead was moved upwards at a speed of 50 mm/min. The force and elongation at breaking point were measured. The following equations were used:

(1) Tensile  strength  (N mm−2) =Force  at  break  (N)Initial  cross-sectional  area  of  sample  (mm2);

(2) Elongation  at  break  (%mm−2) =Increase  in  length  (mm)×100Original  length  (mm)×Cross-sectional  area  (mm2).

#### 2.5.4. Mucoadhesion Study

**Figure 1:**
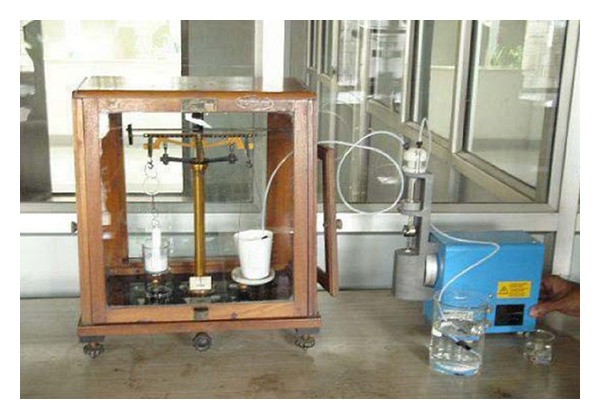
Modified two pan balance used for *in vitro *mucoadhesion studies.

Mucoadhesion of SA and its thiolated derivative-based films were evaluated *in vitro *in triplicate*. In vitro *mucoadhesiveness was measured by modified two pan balance fabricated in our laboratory (Figure 1). The left side of the balance was provided with Teflon blocks attached at the top and in place of the pan at the bottom, and the right side had a receptacle for water.

Mucin films were prepared on cover slips by placing 20 *μ*L of 3% (w/v) porcine mucin in simulated tear fluid (STF, pH 7.4) [16] on a perfectly horizontal surface and air drying the films. During measurement, the films were hydrated with a drop of STF. The cover slips were attached to the Teflon blocks with mucin containing sides facing each other using double sided adhesive tape. The test film was placed between the two cover slips balanced on the left pan of the balance. Films were allowed to adhere to the mucin film and water was promptly added into the receptacle placed on the right pan using a peristaltic pump. Weight in grams of water required to separate the two surfaces was measured and mucoadhesive force was calculated as

(3) $$\begin{array}{c} F=\left(W\times g\right), \end{array}$$

where  *F*  is the mucoadhesion force (mN),  *W*  is the minimum weight required to break the mucoadhesive bond, and *g*  is the acceleration due to gravity (cm/s^2^). Data was analyzed statistically using the *t*-test at a significance level  *P* < 0.05.

#### 2.5.5. Swelling Studies

The swelling index of films of SA and TSA was determined by placing each preweighed film (*W*_1_) in contact with five milliliters of STF in petridish and the dishes were placed in an incubator at 37 ± 0.5°C. At time intervals of 0.5, 1, 2, 3, 4, 6, 8, 10 and 12 h films were gently removed, excess water was carefully blotted away and the swollen films were weighed (*W*_2_). The experiment was repeated thrice and the average weights were used for determination of swelling index as

(4) $$\begin{array}{c} \text{Swelling}\;\text{index}=\frac{\left(W_{2}-W_{1}\right)}{W_{1}}. \end{array}$$

#### 2.5.6. Drug Content

Three randomly selected films of each type were weighed accurately and dissolved in water and/or methanol. The UV absorbances of above solutions were recorded at 286 nm. Concentration of the drug was then extrapolated from the standard curve.

#### 2.5.7. Gas Chromatography

Film samples based on Eudragit were analyzed for residual solvent content by head space analysis using gas chromatography. Acetone and ethanol in a ratio of 80 : 20 diluted appropriately served as standard. The film sample was dissolved in dimethyl formamide and appropriately diluted. The standard and sample solutions (2 mL each) were taken in different 20 mL vials. The vials were sealed and incubated at 105°C with agitation for 20 min; 900 *μ*L of the vapour phase was injected into GC/MS system in a split injection mode (split ratio 1 : 5). Solvents were separated on Thermo Scientific TRACE GC Column (TR-V1 30 m × 0.53 mm ID × 3.0 *μ*m film thickness). The oven temperature was programmed from 35°C held for 5 min to 35°C to 120°C at 15°C/min, held for 1 min to 120°C to 240°C at 40°C/min, and held for 5 min. The total run time was 20 min. The temperature of injector and detector was set at 140°C and 250°C, respectively. The chromatograms were recorded and responses for the major peaks were measured and quantified from calibration curve.

#### 2.5.8. *In Vitro* Drug Release Study

*In vitro *release studies were performed on films using a modification of USP apparatus I with basket, and a stirring speed of 50 rpm was set for the studies. Films were placed in the basket which dipped into 30 mL of dissolution medium (STF) contained in a 100 mL beaker. This beaker was in turn placed into the jar of the dissolution apparatus containing enough distilled water to immerse the beaker up to the level of the release medium. The unit was maintained at 37 ± 0.5°C during dissolution studies and aliquots of 5 mL were withdrawn at each sampling point and replaced with equivalent amount of STF. The amount of drug released was determined using UV-spectrophotometer at 286 nm. Each *in vitro *release study was performed in triplicate and plots of cumulative percent drug released versus time were used to study the release profile.

### 2.6. Preparation and Evaluation of Bilayered Ocular Films

The bilayered films were prepared in two stages: initially films of SA or TSA containing gatifloxacin were prepared in a levelled petriplate by solvent evaporation technique using water as the solvent and glycerine as the plasticizer as described earlier and allowed to dry for 12 h. Without dislodging this layer, above films were carefully covered on the exposed side with a drug containing layer of 20% (w/v) of ERL : ERS (75 : 25) solution in acetone : ethyl alcohol (80 : 20) containing 30% (w/w) of polymer DBP as plasticizer. The solvent was allowed to evaporate for 24 h at 25°C. The bilayered films were gently removed from petridish and cut to result in circular films with an area of 78.5 mm^2^; each containing 2.4 mg of gatifloxacin. Prepared bilayered films were evaluated for thickness, drug content, and drug release using methodology detailed above.

## 3. Results and Discussion

### 3.1. Synthesis and Characterization of Alginate-Cysteine Conjugates

**Figure 2:**
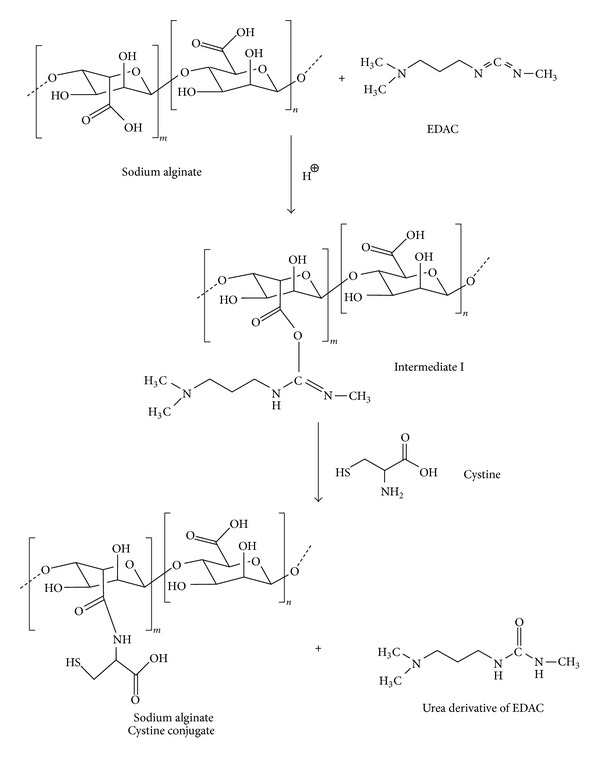
Synthetic scheme for thiolation of alginate.

Figure 2 shows a schematic representation of the EDAC catalyzed amide bond formation between carboxylic acid groups of SA and amine groups of cysteine. The covalent attachment of cysteine to alginate was achieved through formation of amide bond between the primary amino group of amino acid and a carboxylic acid group of the polymer. 1-Ethyl-3-(3-dimethylaminopropyl) carbodiimide (EDAC) catalyzes the formation of amide bonds between carboxylic acids and amines by activation of the later to form an O-acylurea derivative in aqueous medium [17]. The by-product of the reaction is a water soluble urea derivative which can be readily separated from the polymer by dialysis. Thus, in the first step of the present reaction, SA is activated by EDAC at pH 6.0 and an intermediate O-acylurea derivative is formed. In the next step, nucleophilic attack by the nitrogen of the primary amino group of the cysteine results in the formation of the amide bond between –COOH group of the SA and –NH_2_ group of the cysteine and a water soluble urea derivative of EDAC is formed as byproduct. The byproduct and the unreacted cysteine were separated from the thiolated polymer in solution by dialysis. Reaction and dialysis were carried out in the dark to protect the oxidation of –SH groups to –S–S–, catalyzed by light. The efficacy of the purification method described here could be verified, since corresponding control prepared by omitting EDAC during the coupling reaction showed negligible amounts of cysteine after dialysis.

During the synthesis, the oxidation of thiol (–SH) to dithiol (–S–S–) is unavoidable. At pH greater than 5.0 and on exposure to light or higher temperatures during reaction/isolation the degree of oxidation increases. Hence, thiol content was estimated before and after the reduction of thiol groups. Before reduction, a maximum thiol content of 67.05 ± 11.2 *μ*mol/g was found to be present in the derivatized polymer. The thiol content after reduction with NaBH_4_ was found to be 248.80 ± 49.7 *μ*mol/g. This indicates that despite precautions such as synthesis and isolation in dark and dialysis at 10°C, most of the thiol groups are present in oxidized form. The thiol content of polymer was found to be comparable to thiol content of TSA reported in the literature [15].

#### 3.1.1. Fourier Transform Infrared Spectroscopy (FTIR)

**Figure 3:**
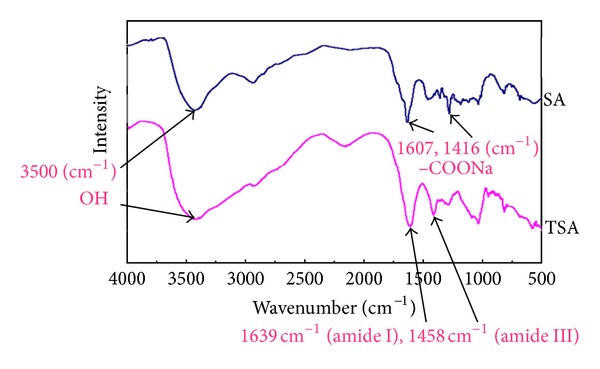
FTIR spectra of SA and TSA.

Figure 3 FTIR spectrum of SA and TSA is compared. The bands of SA appeared at 3500 cm^−1^ for the hydroxyl groups and at 1607 cm^−1^ and 1416 cm^−1^ for the asymmetric –COONa stretching vibration and symmetric –COONa stretching vibration, respectively [18]. The TSA showed distinct amide bond at 1639 cm^−1^ (amide I) and 1458 cm^−1^ (amide III) thereby giving evidence of successful coupling of cysteine via carboxyl groups on polymer backbone.

#### 3.1.2. Rheological Studies

**Figure 4:**
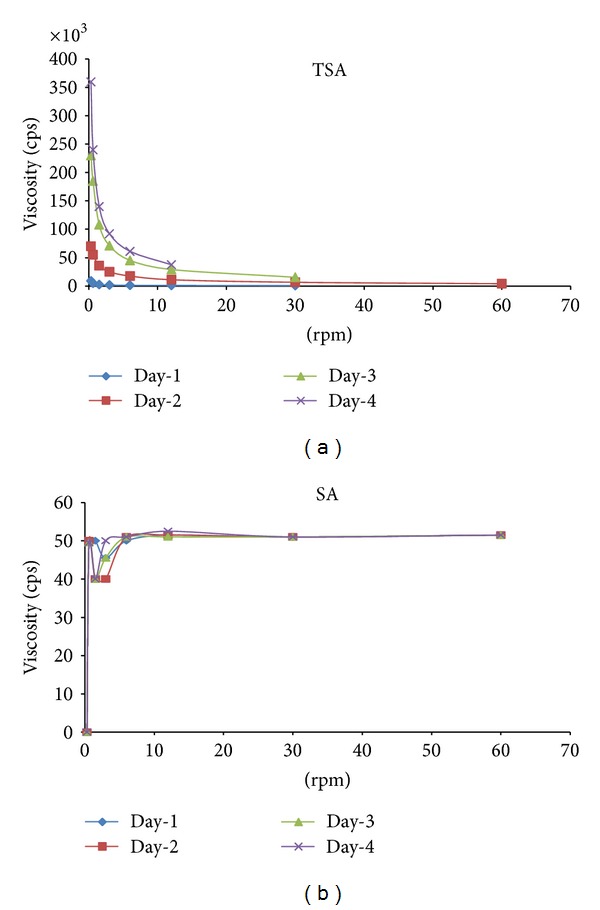
Rheological behaviour of TSA and SA days 1–4.

Native SA solution at the 1% (w/v) concentration level used in the present studies behaved as a Newtonian fluid with nearly constant viscosity of 50 cps throughout the 4-day study period. Change in shear stress (rpm) did not bring about any significant change in viscosity. In contrast TSA solution at the same level (1% (w/v)) had a viscosity of 9000 cps soon after synthesis which increased rapidly during the test period reaching a fortyfold higher viscosity of 3,60,000 cps at the end of the 4 days (Figure 4).

Matrix tablets and microparticulate delivery systems containing thiolated hydrophilic polymers have been shown to be stabilized under physiological conditions by cross-linking via the formation of disulfide bonds. Depending on the pH and the type of thiomer, the thiol groups are oxidized forming inter- and intramolecular disulfide bonds. Consequently, the molecular weight of the polymer increases which can be monitored by a time-dependent increase in viscosity [19]. The results of viscosity studies focusing on this issue carried out with the alginate-cysteine conjugate provided strong support for this theory. The increase in viscosity of the polymeric solution over time can be attributed to disulfide bond formation between alginate chains which also indirectly supports successful thiolation of the polymer.

The thiolated polymer solution also exhibited pseudoplastic behaviour. A decrease in viscosity was observed on increasing shear stress. Mild thixotrophy was also noted.

### 3.2. Evaluation of Ocular Films

The ocular films containing gatifloxacin were successfully prepared by solvent casting technique. Physical characteristics of the films are summarized in Table 1.

All films prepared were translucent and flexible. Folding endurance was found to be more than 300 in each case indicating good flexibility of the films which would contribute towards greater comfort in the eye. The mechanical strength of the TSA films was greater than that of films prepared using native SA. The cross-linking of polymeric chain due to disulphide bond formation may be responsible for the improved mechanical strength. The Eudragit-based films were found to have lower tensile strength and percent elongation values in comparison to the alginate films.The films were uniform with respect to content of drug and did not deviate significantly from the theoretical content.

#### 3.2.1. Mucoadhesion Study

A commonly employed method for assessing the *in vitro *mucoadhesion of particular test substance is the measurement of peak detachment force, the force required to separate mucoadhesives from mucus or mucosa, and was also used in the present studies [20].

**Figure 5:**
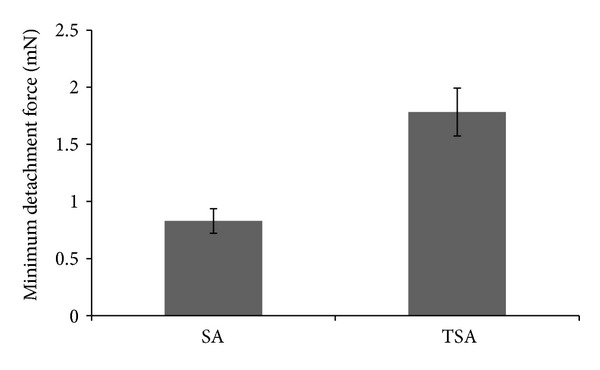
Mucoadhesive strength of films based on SA and TSA.

In the present study film formulations composed of SA and its thiolated derivative were evaluated for *in vitro* mucoadhesive strength. A significant difference in the mean minimal detachment force (MDF) for thiolated and nonthiolated SA was obtained on *t*-test at a significance level of *P* < 0.05 (Figure 5). The MDF for thiolated polymer was greater than two times that for native polymer demonstrating the high affinity of the thiolated alginate for mucosal tissue.

Mucins (molecular weights ranging from 0.5 to 20 MDa) are family of large, extracellular glycoproteins. Mucin molecules consist of proteins which make about 20% of the total mass and are arranged in distinct regions. The region located at the carboxyl and amino terminals has a high amount of cysteine (>10%) which can form disulfide bonds with –SH groups [21]. The resulting disulfide bond is one of the most commonly encountered biological bridges. On the contrary, SA is only able to form noncovalent bonds via ionic interactions and hydrogen bonds within the mucus layer. The disulphide bridging with mucin contributes to the enhanced mucoadhesive strength of TSA.

#### 3.2.2. Swelling Studies

**Figure 6:**
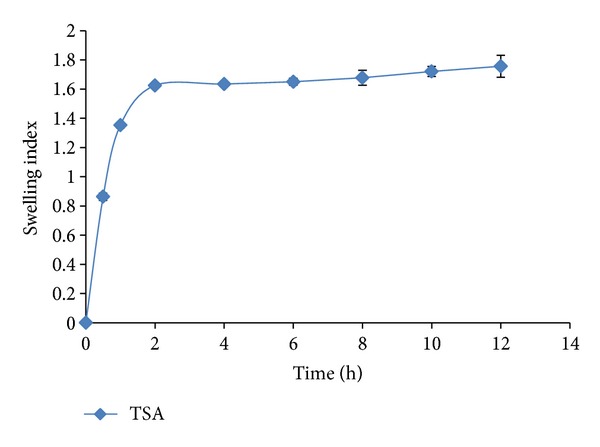
Swelling behaviour of TSA films.

A rapid swelling behavior of mucoadhesive polymers favors the interdiffusion process between the polymer and the mucus layer providing stronger adhesion than in case of poorly swelling polymers. Accordingly, the swelling behavior has an influence on the adhesive properties of polymer [22]. Water-uptake studies were therefore carried out with TSA and native alginate films. The results as shown in Figure 6 demonstrate that films containing native SA completely dissolved in less than 30 min and no weight could be recorded for the films. In contrast, the total amount of STF absorbed by TSA films was 1.8-fold its initial weight. Complete hydration of the film was obtained in less than 60 min. Also no erosion was observed from swollen TSA films, which showed very good cohesive properties. The swollen films were stable for more than 12 h and showed excellent resistance to erosion. This ability of the films prepared using TSA to resist erosion and to remain as a hydrated adhesive layer for prolonged duration is a strikingly beneficial property imparted by thiolation. This will ensure retention of the insert on the ocular surface for the duration of drug release.

#### 3.2.3. Gas Chromatography

Organic solvents like acetone and ethanol have been used for casting films of gatifloxacin. Residual solvents do not provide any therapeutic advantage to the formulation. Thus, there has to be none or minimal concentration of residual solvent content in the film samples so as to not be irritant or toxic to the patient under any circumstances.

Ethanol and acetone were eluted at 4.17 and 4.86 min, respectively. The chromatogram of the film samples in triplicate showed an average solvent content of 8.22 ppm and 22.95 ppm of acetone and ethanol, respectively, which results in an exposure significantly below the permitted limits (less than 5000 ppm per day) [23].

#### 3.2.4. *In Vitro* Drug Release Study

**Figure 7:**
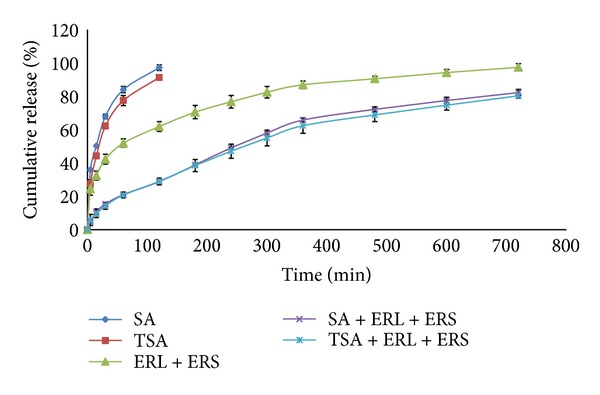
*In vitro* drug release study.

Release profile of gatifloxacin from the films based on SA and TSA are shown in Figure 7. Controlled release properties of polymers are reported to be increased by thiolation due to formation of disulfide bridges within the polymer [19]. This leads to extensive cross-linking of polymer which serves as a barrier to release the drug molecule.

In the present study almost 85% of drug was released from the films based on native SA within 1 h, whereas films based on TSA were found to release about 78% of drug in the same time. Almost 50% of the drug was released from both films within the first 15 min providing a loading dose. However there was no significant difference observed between the cumulative amounts released from both films at each time point when evaluated using *t*-test at a significance level of *P* < 0.05.

This leads to the conclusion that, in the present case, thiolation of SA does not contribute significantly towards improving the controlled release properties of polymer. Sustained release of drug from hydrophilic matrices such as those based on alginate is due to gradual swelling of the polymer starting from the periphery of the matrix and moving inwards and diffusion of the drug through the swollen matrix towards the periphery [24]. The failure of the thiolated polymer to produce significant reduction in release in the present studies is possibly because the studies are on very thin films with large exposed surface leading to rapid swelling and concurrent drug release.

The acrylate-based films showed initial burst release followed by sustained release over the 12 h test period. Almost 70% of drug was released in 12 h. In case of the bilayered films, around 80% of the drug was released over a period of 12 h from both types of inserts. The drug loaded in the bilayered film was estimated with twice a day application in mind. Although release from the films with mucoadhesive layer composed of native alginate was also prolonged, the device may be unable to perform *in vivo* due to erosion of the mucoadhesive layer. In contrast, employing the thiolated derivative as the mucoadhesive component will permit prolonged and superior mucoadhesion, which in turn would allow effective use of the sustained release features of the bilayered insert. Based on the release characteristics of the film, a 12 h period of efficacy may be proposed. *In vivo* studies however are required for establishing the same. There were no significant differences in the *in vitro *release at each time point from both types of bilayered inserts when evaluated using *t*-test at a significance level of *P* < 0.05.

## 4. Conclusion

It was concluded from the above studies that bilayered inserts can provide an effective means of sustained drug delivery to the eye. The alginate layer provides the loading dose as well as helps in anchoring the device intimately with the ocular tissue. The later functionality of the alginate layer was found to be enhanced by thiolation which was found to increase mucoadhesion and offer resistance to erosion by tear fluids. The Eudragit-based rate control layer provides a sustained release of gatifloxacin. Hence, prolonged drug activity and optimal ocular therapy may be achieved by means of the proposed ocular insert. The *in vitro *release profile suggests twice a day application of the device for ocular disease wherein effective concentrations of gatifloxacin can be maintained in ocular tissues.

## Figures and Tables

**Table 1:** Physical characterization of ocular films.

Film formulation	Folding endurance	Tensile strength (N/mm^2^)	% Elongation	Thickness (*μ*m)
SA	328.01 ± 16.24	95.3	28.24	54.2 ± 3.1
TSA	332.1 ± 15.26	129.2	29.93	61.8 ± 2.0
ERL + ERS	480.4 ± 10.28	26.85	157.6	238.4 ± 3.2
SA + ERL + ERS	—	—	—	276.695 ± 2.0
TSA + ERL + ERS	—	—	—	270.235 ± 2.6
